# Case of Spontaneous Rupture of the Uterus in the Third or Fourth Month of Pregnancy

**Published:** 1846-10

**Authors:** 


*Case of spontaneous rupture of the Uterus in the third or fourth month of pregnancy. Congenital absence of muscular wall of Uterus on ons side, of Fallopian tube and ovary, and of the os Tincæ. Communicated in a letter to Prof. Hamilton, by* ——— ———, *M. D.*

*Aug.* 7, 1846.

*Dear Sir :* I have recently made an autopsy which I think of sufficient interest to report to you.

Miss ———, æt. 17, was seized suddenly with pains resembling colic, accompanied with vomiting and faintness. A neighbor being called in prescribed as for colic, and the girl partially revived, but soon became faint again, and died.

*Autopsy*, 24 *hours after decease.*—Cavity of abdomen filled with coagulated blood, surrounding a fœtus of the third or fourth month. Uterus ruptured along its right side, from the fundus to near the neck. Left ovary and Fallopian tube entirely wanting. Right ovary and Fallopian tube were present, but the latter entered the uterus nigh to the neck, and the ovary was correspondingly lower than natural.

The uterus appeared to be developed to the size of the third or fourth month of gestation. The placenta was attached near the fundus upon the left side. On the side of the uterus where the rupture occurred, the walls of the organ were extremely thin. At the time of rupture there seemed to be nothing but the peritoneum, and in the immediate neighborhood the friability was such, that it was readily torn by the fingers with slight force. On the left side the walls were of the usual thickness, but did not present the common fibrous appearance. On examination of the neck no aperture could be found, nor was there any trace of an *os tincæ.* The neck resembled a tendon in appearance, but of less density. There was no communication whatever between the cavity of the uterus and the vagina.

I have retained the parts, and will embrace some opportunity to submit them to your inspection. The case is certainly one of interest as connected with the inquiry how the spermatic secretion was transmitted so as to have effected fecundation. It would seem that it disproves incontestibly the necessity of the reception of this secretion into the uterine cavity, and from thence into the Fallopian tube; but the question remains, how *was* the necessary contact accomplished? I see no other method of explanation except by the old doctrine of absorption.

With much esteem, I remain yours,

——— ———.

Dr. F. H. Hamilton.

*Buffalo*, Sept. 6, 1846.

Dr. Flint,—Dr. ———, (for obvious reasons names are suppressed,) a few days since brought to me the ruptured uterus and fœtus described in his letter, and in his presence, with Dr. Mixer, I have examined the whole carefully.

The uterus is torn near the attachment of the right broad ligament—walls on this side excessively thinned, so much so in some places as to leave little else than peritoneum,—opposite side of uterus about $\frac{1}{3}$ of an inch in thickness, and of usual consistence. Length of body of uterus, 3 inches—breadth $2\frac{1}{2}$ inches; cervix uteri 2 inches long, firm and broad at base, but gradually and regularly tapering to a thin, flattened extremity, covered by a smooth mucous membrane. Upon a careful dissection of the cervix, longitudinally and transversely, no channel, or line, or cicatrix, indicating the previous existence of an os tincæ could be discovered. The structure is rather more dense than that of the uterus in its natural state, but its density is uniform—it is not scarred or corrugated, nor does it present any evidence of its being a result of disease. The mucous coat which covers it, lies free, and can be as easily removed as such membranes usually may be.

The right Fallopian tube terminates at about the middle of the right side of uterus—right ovarium is larger than natural, but apparently healthy. On the left side there is no broad ligament, nor Fallopian tube, nor ovarium, but the peritoneum covers it as smoothly as in front.

Dr. ——— adds to what has been said in his letter, that the girl was always healthy—had menstruated 3 years, or until the last 3 months, regularly.

The whole was laid before the Buffalo Medical Association for their examination, at its last meeting, (Sept. 2d,) and the records of the society certify substantially to the same as above.

Through the politeness of Dr. ———, the uterus, &c., is now the property of the Buffalo Medical College, and may at any time be seen by medical gentlemen. F. H. HAMILTON.

*Ibid.*

